# Mesenchymal stromal cell therapy in epidermolysis bullosa: current perspectives and future directions

**DOI:** 10.3389/fcell.2025.1703109

**Published:** 2025-11-17

**Authors:** Twan Sia, Rosita Primavera, Max R. Johnson, Haripriya Sai Dukkipati, Joyce M. C. Teng, Avnesh S. Thakor

**Affiliations:** 1 Center for Interventional Radiology Innovation at Stanford (IRIS), Stanford University School of Medicine, Palo Alto, CA, United States; 2 Stanford University School of Medicine, Department of Dermatology, Palo Alto, CA, United States

**Keywords:** epidermolysis bullosa, mesenchymal stem cells, mesenchymal stromal cells, extracellular vesicles, therapeutics

## Abstract

Epidermolysis bullosa (EB) is a group of inherited mucocutaneous disorders. Mesenchymal stromal cells (MSCs) are non-hematopoietic self-renewing, multipotent cells that are a promising therapeutic avenue for EB, given their ability to home to injury, low immunogenicity, and demonstrated wound-healing, anti-fibrotic, and pro-collagen effects. This review article synthesizes the current literature and advancement on MSC therapy in EB, and highlights the potential to optimize their use, including exploring MSC-derived extracellular vesicles as a potential cell-free therapy. Innovative delivery methods can also improve the accessibility and effectiveness of MSC therapies by providing localized treatment, minimizing systemic side effects, and increasing patient comfort.

## 1 Introduction

Epidermolysis bullosa (EB) encompasses a heterogeneous group of inherited skin disorder (genodermatoses) characterized by extreme skin fragility, where even minimal trauma can lead to mucocutaneous blistering, chronic wounds, and scarring. The severity of EB varies widely across its four major subtypes: epidermolysis bullosa simplex (EBS), junctional epidermolysis bullosa (JEB), dystrophic epidermolysis bullosa (DEB), and kindler epidermolysis bullosa (KEB) (Bardhan et al., 2020). The most severe forms of EB, particularly DEB, are associated with systemic complications, including generalized blistering, involvement of internal epithelialized organs, an elevated risk of aggressive skin cancer, and ultimately, early mortality (Bardhan et al., 2020). Each EB subtype arises from mutations in specific genes that play critical roles in skin integrity. For instance, DEB is caused by mutations in the *COL7A1* gene, which encodes type VII collagen (C7), a crucial component of anchoring fibrils that stabilize the dermal-epidermal junction (Bardhan et al., 2020). Disruption of C7 function in DEB leads to severe blistering and delayed wound healing, resulting in the increased morbidity and mortality associated with the disease (Bardhan et al., 2020).

Traditional management of EB is largely supportive and symptomatic, including meticulous wound care (Pope et al., 2012; Pabón-Carrasco et al., 2024), pain control (Goldschneider et al., 2014), psychosocial support (Martin et al., 2019), physical and occupational therapy to maintain mobility and function (Chan et al., 2019; Weisman et al., 2021). Recent US Food and Drug Administration approvals of Oleogel-S10 for DEB and JEB and the gene therapy beremagene gerperpavec (Vyjuvek) for DEB have provided some relief, though neither offer a definitive cure. Oleogel-S10 is a topical gel consisting of 10% birch triterpenes in sunflower oil, which functions through antimicrobial, anti-inflammatory, and pro-wound healing properties (Kern et al., 2023).

Mesenchymal stromal cells (MSCs), known for their multipotency, self-renewal capacity, and non-hematopoietic origins, are emerging as a promising therapeutic avenue for EB. Their multifaceted therapeutic potential has been demonstrated in various disease models, where their immunomodulatory, wound-healing, anti-fibrotic, and pro-collagen effects are harnessed (Golchin et al., 2019; Galderisi et al., 2022). MSCs have low immunogenicity and possess the ability to home to injury sites, which further supports their potential as a treatment for the systemic nature of severe EB subtypes like DEB. In both preclinical and early clinical studies, MSCs have shown promising safety profiles and preliminary efficacy in mitigating the symptoms of EB, suggesting that MSC therapy could become an important part of future treatment regimens.

This narrative review will delve into the current literature on the application of MSCs in EB. It will explore the underlying mechanisms by which MSCs exert their therapeutic effects, summarize the clinical outcomes from MSC therapies, and discuss strategies to enhance their administration and efficacy in treating EB.

## 2 Mesenchymal stromal cell therapy in EB

MSCs are non-hematopoietic, self-renewing, multipotent cells that have been investigated in several challenging clinical applications, including myocardial infarction, graft-versus-host disease, and Crohn’s disease. MSCs have been isolated from various sources, with the most common being bone marrow (BM-MSCs), umbilical cord (UC-MSCs), and adipose tissue (AD-MSCs). Several factors make MSCs an appealing therapeutic option. Notably, they possess a strong homing capacity to injured tissues that release high mobility group box 1 (HMGB1) (Tamai et al., 2011). In fact, a unpublished phase II trial of intravenous modified HMGB1 alone in participants with RDEB preliminarily showed improved wound healing (Hou et al., 2023). Homing of MSCs can be further enhanced for targeted delivery through various strategies, including cell surface or genetic engineering, *in vitro* priming, or magnetic guidance (Ullah et al., 2019). In addition, MSCs generally express low levels of human leukocyte antigen (HLA)-I and lack expression of HLA-II, allowing for allogenic transplantation with a reduced risk of rejection (Zhou et al., 2021). Rejection may still occur when allogeneic MSCs are used, and MSCs may still be cleared by host innate immune mechanisms.

Despite their mesodermal origin and differentiation potential, the tissue repair capacity of MSCs through differentiation and engraftment remains relatively weak. However, emerging evidence suggests that the therapeutic benefits of MSCs are primarily mediated through their paracrine effects. MSCs secrete a variety of soluble factors and can also deliver therapeutic molecules *via* extracellular vesicles (EVs). This diverse secretome includes proteins, growth factors, enzymes, and various RNAs. These paracrine factors have been shown to exert multiple downstream effects, such as cytoprotection, neovascularization, anti-inflammation, anti-fibrosis, and tissue repair (including anti-apoptotic and pro-mitotic effects) (Ghannam et al., 2010; Gnecchi et al., 2016).

Only a few MSCs- or MSC-derived-products have reached clinical implementation for limited indications. Standardization represents a big challenge. The current literature is varied in terms of MSC source and culturing/processing techniques, which makes reproducibility difficult. For clinical implementation, additional variability is introduced when considering differences in donors, cryopreservation procedures, treatment protocols, *etc.* These small differences can critically alter biological properties, and thus, treatment efficacy. There is also currently no global consensus on release assays, which are used to confirm potency, purity, and biological activity prior to patient administration. Scalability, especially without compromising standardization, represents another challenge to clinical implementation of MSCs. Future work creating consensus protocols that are efficient and accessible are paramount to overcoming these challenges (Lukomska et al., 2019; da Silva et al., 2025).

### 2.1 Preclinical studies

**FIGURE 1:**
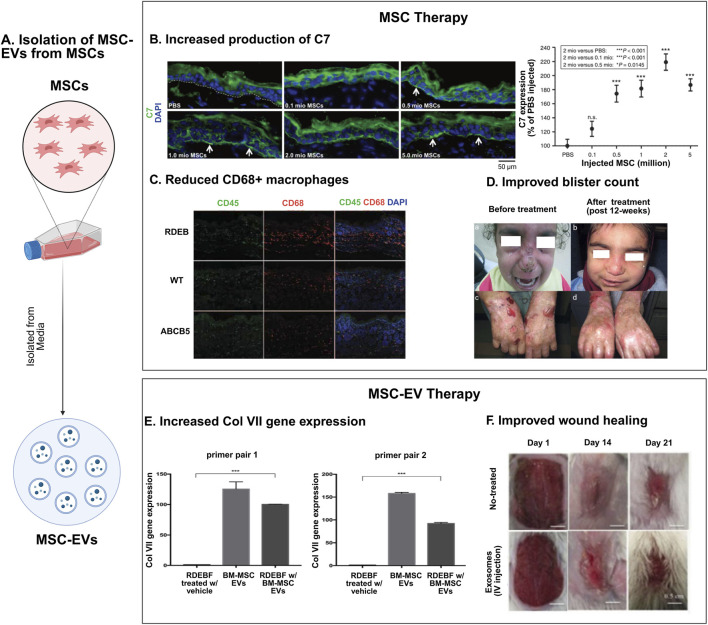
Mesenchymal stromal cell therapies in epidermolysis bullosa. **(A)** Schematic representation of MSC-extracellular vesicle (MSC-EV) isolation from MSCs. **(B–D)** MSC therapy for EB: MSCs are effective in treating EB, exerting several therapeutic effects, such as increasing production of type VII collagen protein (C7) (reproduced from Kühl et al., 2015) **(B)**, showing immunosuppressive effects through a reduction in CD68^+^ macrophages (reproduced from Webber et al., 2017) **(C)**, and reducing blister count in EB patients (reproduced from El-Darouti et al., 2016) **(D)**. **(E, F)** MSC-EV therapy: MSC-EVs have demonstrated increased expression of collagen gene Col VII (reproduced from McBride et al., 2018) **(E)**, and improved wound healing in murine models of EB (reproduced from Hu et al., 2016) **(F)**.

In preclinical studies of EB, MSCs have been primarily investigated as a therapeutic approach for recessive dystrophic epidermolysis bullosa (RDEB) (Figure 1). The earliest study investigating the therapeutic effect of MSCs in murine models was conducted by Tolar et al., in 2009, which showed that infusion of BM-MSCs did not prolong the lifespan of *Col7a1* ^*−/−*^ mice (Tolar et al., 2009). The following studies of BM-MSCs in murine models of RDEB investigated more granular endpoints (Kühl et al., 2015). Kühl et al. demonstrated that intradermally injected BM-MSCs secreted functional collagen type VII (C7) at the DEJ in C7-hypomorphic murine models in a dose-dependent fashion. At the highest dose they tested (2 × 10^6^ MSCs/cm^2^ of targeted skin), they observed enhanced wound regeneration, reduced inflammation, and improved granulation tissue formation (Figure 1B). Additionally, BM-MSC injection also appeared to restore some immature anchoring fibrils at 12 weeks. Unfortunately, due to the lethality of the DEB phenotype in these mice, as well as possible immune rejection of human MSCs given the immunocompetence of these mice models, longer-term follow-up was not possible (Kühl et al., 2015).

Building on this work, Ganier et al. investigated the potential long-term effects of intradermal injection of the same dose of BM-MSCs in an immunodeficient RDEB xenograft murine model. Their study reported C7 production and anchoring fibril formation up to 6 months post-treatment, comparable to healthy skin. Ganier et al. hypothesize that this long-term effect might be attributed to the immunodeficient nature of their model, which prevented immune rejection of the human MSCs, thus raising the question if autologous MSCs may be advantageous to allogeneic MSCs (Ganier et al., 2018). In comparison, MSCs in the 2015 Kühl et al. study underwent apoptosis within 28 days, so long term benefits of MSCs would not be expected (Kühl et al., 2015). Still, isolating autologous MSCs in clinical practice, particularly from invasive sites such as bone marrow, would be exceedingly challenging in patients with RDEB due to the fragility of their skin.

Human dermal MSCs (D-MSCs), identified by ATP-binding cassette member B5 expression (ABCB5) which mediates cell cycle quiescence (Schatton et al., 2015; Niebergall-Roth et al., 2022), have also been explored in preclinical RDEB mouse models. Webber et al. generated an immunodeficient *COL7A* ^*−/−*^ mouse model using the CRISPR/Cas9 system in immunodeficient embryos. Systemic administration of ABCB5^+^ D-MSCs into RDEB model mice resulted in improved survival. However, skin biopsies of long-term surviving mice did not show an increase in C7 levels, nor were ABCB5+ D-MSCs detected. This finding supported the hypothesis that ABCB5+ D-MSCs might provide therapeutic benefit in RDEB through a C7-independent pathway in contrast to previous work on BM-MSCs. They suggested that the improved survival may be attributed to the immunosuppressive properties of ABCB5+ D-MSCs, as evidenced by a decrease in CD68^+^ macrophages 48 h after treatment with ABCB5+ D-MSCs (Figure 1C) (Webber et al., 2017). Skin biopsies that tested for C7 were taken on day 67, and earlier expression of C7 that decreased over time is unlikely, as C7 has a relatively long half-life of about 1 month (Kühl et al., 2016), though cannot be ruled out. Conversely, it is also possible that BM-MSCs operate through a C7-independent mechanism of action. As such, the precise mechanism by which MSCs exert their therapeutic effects in RDEB remains unclear, especially if these mechanisms differ based on the tissue source of the MSC (Webber et al., 2017).

Further comparative studies of ABCB5+ D-MSCs and BM-MSCs in RDEB mouse models revealed that ABCB5+ D-MSCs exhibited superior homing to wound sites compared to BM-MSCs, likely mediated by increased expression of transcription factor HOXA3 in ABCB5+ D-MSCs (Riedl et al., 2021). Therefore, Riedl et al.’s findings suggest that ABCB5+ D-MSCs may be preferential for the treatment of EB given its high burden of cutaneous wounds.

UC-MSCs were also investigated in the treatment of RDEB in mouse models. Lentiviral transduction was used to overexpress C7 in UC-MSCs, and intradermal administration of these lentiviral transduced UC-MSCs into an immunocompromised mice model with human RDEB grafts resulted in C7 deposition and subsequent anchoring fibril formation to normal levels at the DEJ (Petrova et al., 2020). However, since the study did not describe the effects of intradermal administration of non-lentiviral transduced UC-MSCs, it is unclear whether lentiviral transduction provides a clinically relevant therapeutic benefit. They also provided preliminary data showing that their lentiviral-transduced UC-MSCs could not be delivered effectively systemically through tail vein injections, as the MSCs did not localize to skin sites (Petrova et al., 2020). Given that tail vein injections have not been utilized in studies with BM-MSCs or ABCB5+ D-MSCs, it is difficult to compare their systemic delivery potential directly.

Notably, MSCs have been isolated from the blister fluid of patients with RDEB (Bf-MSCs). Bf-MSCs modified to express C7 and applied *via* either cell sheets or intra-blister injections showed improvement of C7 expression in murine models of RDEB. Thus, Bf-MSCs represents a potentially advantageous source of autologous MSCs, as other modes of collecting MSCs are invasive and particularly painful to patients with EB. Additionally, drainage of blister fluid can be part of the routine care for patients with RDEB, allowing for the collection of large amounts of Bf-MSCs (Kikuchi et al., 2023).

While MSC-based therapies have shown promise for RDEB, other subtypes of EB have yet to be explored in preclinical MSC studies. Though different types of EB share similar pathomechanisms, many are likely subtype specific. Thus, despite the demonstrated therapeutic efficacy in various cutaneous diseases beyond RDEB, further studies of MSCs in non-RDEB models of EB are needed to confirm whether MSCs may also offer therapeutic benefits for other clinical subtypes of EB.

### 2.2 Clinical trials

#### 2.2.1 Earlier clinical trials

The primary literature on the use of MSCs in patients with EB is summarized in Table 1. Previous work consists mainly of preliminary data in case reports or Phase I/II trials. These studies vary widely in terms of MSC dosage, dosing schedule, route of administration, and the origin of MSCs used. However, consistent with preclinical studies, the majority of clinical research has focused on the RDEB subtype. While the earliest case report by Conget et al. employed intradermal administration of MSCs (Conget et al., 2010), later studies have shifted to intravenous administration of MSC. Given that localized administration of MSCs demonstrated localized benefit, future studies shifted to systemic delivery to see if there would be systemic effects, as EB can have multisystemic involvement. Early clinical studies also focused on BM-MSCs, with more recent studies administering AD-MSCS (Maseda et al., 2020), ABCB5+ D-MSCs (Kiritsi et al., 2021; Dieter et al., 2023; Niebergall-Roth et al., 2023), and umbilical cord blood-derived MSCs (UCB-MSCs) (Lee et al., 2021). However, omics-based functional analysis has demonstrated that the regenerative signature of MSCs is source-specific (Ganguly et al., 2023). Comparing the safety and efficacy of MSCs from different tissue origins is challenging due to variations in dosing concentrations, schedules, and clinical endpoints. To date, no head-to-head clinical trials have been conducted to directly compare MSCs from different tissue origins.

**TABLE 1:** Previously reported clinical trials of mesenchymal stromal cells and multilineage-differentiating stress-enduring (MUSE) cells in patients with epidermolysis bullosa.

Therapy	Administration route	Patients	Study design	Outcome	References (identifier number, if available)
Allogeneic BM-MSCs (0.5 × 10^6^ cells)	Intradermal	RDEB (Adult, n = 1; pediatric, n = 1)	Case report	Restoration of COL VII along the basement membrane and DEJ up to 4 months post-treatment Localized re-epithelialization of chronic ulcerated skin	Conget et al. (2010)
Allogeneic BM-MSCs (1–3x10^6^ cells/kg, on day 0, 7, and 28)	Intravenous	RDEB (pediatric, n = 10).	Phase I/IIa	Treatment emergent adverse events included DMSO odor, nausea, abdominal pain, and bradycardia. No adverse events were severe Improvement in Birmingham EB Severity Score and global severity score questionnaire	Petrof et al., 2015 (EudraCT 2012-001394-87; ISRCTN46615946)
BM-MSCs (unspecified dosage, from parent) (n = 7) with or without cyclosporine 5 mg/kg (n = 7)	Intravenous	RDEB (Adult, n = 1; pediatric, n = 13)	Phase II	Decreased rate of new blister formation and increased healing of new blisters Increased number of anchoring fibrils	El-Darouti et al. (2016)
Allogeneic BM-MSCs (2 × 10^6^ cells/kg on day 60, 100, 180) following bone marrow transplant with post-transplant cyclophosphamide	Intravenous	RDEB (pediatric, n = 10)	Phase I/II	No adverse events were attributable to MSC infusions	Ebens et al., 2019 (NCT02582775)
Allogeneic AD-MSCs (10^6^ cells/kg, every 21 days x3 doses)	Intravenous	RDEB (pediatric, n = 1)	Case report	Large improvement in Birmingham EB Severity Score and EBDASI maximally at 6-, and 9-month post-treatment respectively Modest improvement in Visual Analog Scale pain, Leuven Itch Scale, and quality of life (EuroQoL-5D)	Maseda et al. (2020)
Allogeneic BM-MSCs (2–4x10^6^ cells/kg, every 14 days x2 doses)	Intravenous	RDEB (adult, n = 9)	Phase I/II	No serious treatment emergent adverse events for 12 months Improvement in EBDASI, Quality of Life in EB score, and Leuven Itch Scale	Rashidghamat et al., 2020 (NCT02323789; EudraCT 2014-004500-30)
Allogeneic ABCB5+ D-MSCs (2 × 10^6^ cells/kg, on day 0, 17, and 35)	Intravenous	RDEB (adult, n = 7; pediatric, n = 9)	Phase I/IIa	Adverse events were mild lymphadenopathy (n = 1) and hypersensitivity reactions (n = 2) Improvement in EBDASI, Instrument for Scoring Clinical Outcome of Research for EB clinician score, and pruritus Increased wound closure and decreased rate of new wounds	Kiritsi et al., 2021; Dieter et al., 2023; Niebergall-Roth et al., 2023 (NCT03529877; EudraCT 2018-001009-98)
Allogeneic UCB-MSCs (1–3x10^6^ cells/kg, every 14 days x3 doses)	Intravenous	RDEB (adult, n = 4; pediatric, n = 2)	Phase I/IIa	No serious treatment-emergent adverse events Improvements in Birmingham EB Severity Score, body surface area involvement, Visual Analog Scale pain, quality of life (QOLEB), and blister count	Lee et al., 2021 (NCT04520022)
Allogeneic MUSE cells (CL2020	Intravenous	DEB (adult, n = 4; pediatric, n = 1)	Phase I/II	Mild, self-limited adverse events Improvement in ulcer size and visual analogue score for pain, but not for itch or quality of life	Fujita et al., 2021a (JapicCTI-184563)

Abbreviations: ABCB5+ D-MSCs, ABCB5+ dermal-derived mesenchymal stromal cells; AD-MSCs, adipose-derived mesenchymal stromal cells; BM-MSCs, bone marrow-derived mesenchymal stromal cells; DEJ, dermal-epidermal junction; EB, epidermolysis bullosa; EBDASI, EB Disease Activity and Scarring Index; RDEB, recessive dystrophic epidermolysis bullosa; UCB-MSCs, umbilical cord blood-derived mesenchymal stromal cells; MUSE, multilineage-differentiating stress-enduring; DEB, dystrophic epidermolysis bullosa.

The clinical endpoints investigated in each clinical trial are highly heterogeneous and have therefore been summarized in Table 1. Despite this variability, Phase ll studies have reported promising, albeit transient, clinical benefits in patients with RDEB. One key area of interest is the effect of MSC therapy on the expression of C7, which is deficient or defective in RDEB due to *COL7A1* mutations. Some studies, including those using BM-MSCs (Conget et al., 2010) and UCB-MSCs (Lee et al., 2021), reported an increase in C7 levels upon skin biopsy following MSC therapy. However, other studies found no significant change in C7 expression post-therapy with BM-MSCs (Petrof et al., 2015; El-Darouti et al., 2016; Rashidghamat et al., 2020). In addition, several studies did not examine skin biopsies for changes in C7 or anchoring fibril formation (Maseda et al., 2020; Kiritsi et al., 2021). The mixed results may be due to the transient effects of MSCs on C7, as biopsies were taken at varying times post-treatment, or it could indicate that MSCs have a limited impact on C7 restoration. Additionally, there is a possibility that MSCs of certain origins may exhibit different capabilities of increasing C7 levels. Furthermore, part of the heterogeneity may be due to different genotypes of RDEB included in each study.

Importantly, MSCs have been remarkably well tolerated in EB clinical trials, with no severe adverse events directly attributed to MSC therapy in several studies (Conget et al., 2010; El-Darouti et al., 2016; Maseda et al., 2020; Rashidghamat et al., 2020; Lee et al., 2021). However, in a BM-MSC trial, Petrof et al. reported minor adverse events in 10 patients, including DMSO odor (2 cases, 20%), mild nausea (2 cases, 20%), and abdominal pain and bradycardia (2 cases, 20%), all of which resolved without intervention or discontinuation of therapy (Petrof et al., 2015). In a single-arm trial of ABCB5+ D-MSC involving 16 patients, Kiritsi et al. reported mild lymphadenopathy in one patient (6.25%) and hypersensitivity reactions in two patients (12.5%) that were severe enough to warrant withdrawal from treatment. The hypersensitivity reactions resolved without further complications (Kiritsi et al., 2021). In comparison, an open-label extension trial of beremagene gerperpavec (Vyjuvek) in patients with DEB have shown mild-moderate adverse events in a majority of patients (74.5%), though none led to treatment discontinuation (Marinkovich et al., 2025).

#### 2.2.2 Active clinical trials

According to ClinicalTrials.gov, ongoing trials of MSCs for the treatment of EB are summarized in Table 2. While earlier clinical trials focused on RDEB, current trials are recruiting for other subtypes of EB, such as JEB and DDEB, often without requiring genotyping. Consistent with previous work, upcoming clinical trials continue to focus on ABCB5+ D-MSCs and AD-MSCs instead of BM-MSCs. ABCB5+ D-MSCs are favored for their superior skin homing capabilities, attributed to *HOXA3* gene expression, and their potential to secrete C7 (Riedl et al., 2021). AD-MSCs are more abundantly accessible and are considered to offer benefits comparable to BM-MSCs, though differences in gene expression profiles, secreted factors, and disease-specific data remain notable (Strioga et al., 2012). Among these proposed clinical trials is the first double-blind, randomized, placebo-controlled trial of MSCs (NCT05464381), representing the most compelling clinical evidence to date. In addition, future clinical trials are investigating topical applications of MSCs, including the use of hydrogel sheet (NCT02579369; NCT03183934) and other topical delivery methods (NCT05157958). These trials will provide critical insights into the safety and efficacy of MSC therapies for EB subtypes beyond RDEB and evaluate the potential advantages of topical MSC delivery.

**TABLE 2:** Ongoing or unpublished clinical trials of MSCs and MSC-EVs in patients with epidermolysis bullosa registered in the United States (ClinicalTrials), United Kingdom (ISRCTN), Europe (EudraCT), and Japan (UMIN-CTR).

Therapy	Administration route	Patients	Study design	Identifier number
MSCs				
Allogeneic ABCB5+ D-MSCs (allo-APZ2-OTS; unspecified dosage)	Intravenous	Pediatric and adult participants with RDEB or JEB (estimated enrollment n = 74)	Phase III (double-blind, placebo-controlled, randomized)	NCT05464381
Allogeneic hematopoietic SC transplant followed by UCB-MSC or BM-MSC (unspecified dosage)	Intravenous	Pediatric and adult participants with severe EB (estimated enrollment n = 32)	Phase II	NCT01033552
Allogeneic cord blood transplant with MSC co-infusion (unspecified origin and dosage)	Intravenous	Pediatric participants (estimated enrollment n = 2)	Phase II	EudraCT 2012-000605-72
Allogeneic adipose MSCs (ALLO-ASC-DFU; unspecified dosage)	Topical (hydrogel sheet)	Pediatric and adult participants with DEB (estimated enrollment n = 5)	Phase I/II and open-label extension	NCT02579369; NCT03183934
Allogeneic MSCs (ALLO-ASC-SHEET; unspecified origin and dosage)	Topical (hydrogel sheet)	Pediatric and adult participants with DEB (estimated enrollment n = 6)	Phase II	NCT05157958; UMIN000028366
Allogeneic AD-MSC (unspecified dosage)	Intradermal	Pediatric and adult participants with EB (estimated enrollment n = 15)	Phase I/II	EudraCT 2020-002936-55
Allogeneic UC-MSC (2–3 × 10^6^ cells/kg, on day 0 and 14, followed by 1–1.5 × 10^6^ cells/kg)	Intravenous	Pediatric participants with RDEB (estimated enrollment n = 36)	Phase I (double-blind, placebo-controlled, crossover)	ISRCTN14409785
Haploidentical BM-MSCs; 2–3 × 10^6^ BM-MSCs/kg weekly for 3 weeks	Intravenous	Pediatric participants with RDEB (estimated enrollment n = 9)	Phase I/II	NCT04153630; EudraCT 2017-000606-37
Allogeneic ABCB5+ D-MSCs (allo-APZ2-OTS); (unspecified dosage)	Intravenous	Pediatric and adult participants with RDEB (estimated enrollment n = 74)	Phase III (double-blind, placebo-controlled, randomized)	NCT05838092
MSC-EVs				
BM-MSC-EVs (exosomes, unspecified dosage)	Topical	Pediatric and adult participants with DEB (estimated enrollment n = 10)	Phase I/IIa	NCT04173650

Abbreviations: ABCB5+ D-MSCs, ABCB5+ dermal-derived mesenchymal stromal cells; BM-MSCs, bone marrow-derived mesenchymal stromal cells; BM-MSC-EVs, bone marrow-derived mesenchymal stromal cell extracellular vesicles; DEB, dystrophic epidermolysis bullosa; DEJ, dermal-epidermal junction; EB, epidermolysis bullosa; JEB, junctional epidermolysis bullosa; MSCs, mesenchymal stromal cells; MSC-EVs, mesenchymal stromal cell extracellular vesicles; RDEB, recessive dystrophic epidermolysis bullosa; UCB-MSCs, umbilical cord blood-derived mesenchymal stromal cells.

### 2.3 Multilineage-differentiating stress-enduring cells

Multilineage-differentiating stress-enduring (MUSE) cells are a subpopulation of MSCs with a stage-specific embryonic antigen (SSEA)-3 marker. Like MSCs, MUSE cells are non-immunogenic and exhibit several mechanisms of repair, including cell replacement and paracrine effects on tissues (Young, 2018). In addition, MUSE cells can be isolated from various MSC sources, primarily bone marrow (Kuroda et al., 2010), but also from dermal tissue (Tian et al., 2017), adipose tissue (Yamauchi et al., 2017), and peripheral blood (Sato et al., 2020).

Several properties make MUSE cells particularly promising for potential therapy in EB. As their name suggests, MUSE cells exhibit high stress tolerance, due to the expression of 14-3-3 proteins and serpins. The 14-3-3 proteins protect MUSE cells against stress-induced apoptosis, while serpins, which are uniquely expressed in MUSE cells but not in conventional MSCs, inhibit apoptosis mediators, such as caspases and trypsin (Li et al., 2022). Additionally, MUSE cells demonstrate greater multipotency than MSCs. Unlike non-MUSE cells within the MSC population, MUSE cells can proliferate even in single-cell suspension (Kuroda et al., 2010; Dezawa, 2016). In an animal model of acute myocardial infarction, MUSE cells exhibited an approximately 2.5-fold greater reduction in infarction size and 2.0-fold greater cardiac output improvement compared to MSCs (Yamada et al., 2018).

MUSE cells have been investigated in a murine model of JEB. In adult collagen type XVII (C17) knockout mice with simulated wounds, intravenously administered MUSE cells showed greater homing potential to wounds compared to non-MUSE BM-MSCs, with greater integration of C17 (Fujita et al., 2021a). CL2020 is a MUSE cell-based product that has been investigated in clinical trials for several disorders, including acute myocardial infarction (Noda et al., 2020), stroke (Niizuma et al., 2023), amyotrophic lateral sclerosis (Yamashita et al., 2023). CL2020 administration to a murine JEB model demonstrated significant deposition of C7 and C17 compared to vehicle. This persisted to 6 months after treatment, however, there was no difference in survival rate of CL2020-treated *versus* vehicle-treated mice (Fujita et al., 2021a).

In an open-label Phase I/II study involving five patients with DEB, a single infusion of CL2020 resulted in mild, self-limited adverse effects. Following the infusion with CL2020, there was a significant decrease in ulcer size and improvement in pain scores, though no notable changes in quality of life or itch were observed. A skin biopsy from one patient did not show an increase in C7 or anchoring fibrils (Fujita et al., 2021b). Further research is needed to assess the long-term tolerance and efficacy of MUSE cell-based therapies in EB.

### 2.4 Mesenchymal stromal cell-derived extracellular vesicles

MSCs secrete extracellular vesicles (EVs), including exosomes and microvesicles, which encapsulate microRNA (miRNA), messenger RNA (mRNA), and proteins. Given the paracrine mechanism of MSCs, EVs have garnered interest as a potential cell-free therapeutic alternatives to MSC, offering the possibility of delivering therapeutic benefits through their bioactive cargo (Hade et al., 2022). *In vitro* experiments on RDEB fibroblasts have identified two primary therapeutic mechanisms of MSC-derived EVs (MSC-EVs): 1) aiding in the extracellular transport of C7, and 2) delivering mRNA encoding for C7, which enables RDEB fibroblasts to translate *COL7A1* and subsequently synthetize the C7 alpha-1 chain (McBride et al., 2018) (Figure 1E). Despite these promising findings, the literature on MSC-EVs in EB remains limited. It is yet to be determined whether additional EV cargos contribute to therapeutic outcomes in a C7-independent manner. Furthermore, it remains unclear whether EVs derived from MSCs of different tissue origins carry distinct therapeutic cargoes or if enhancing EV release from MSCs could amplify their therapeutic efficacy.

Other insights into the potential of EVs in treating EB can be drawn from studies on skin wound healing. For instance, systemic administration of AD-MSC-EVs in murine models of skin incision accelerated wound healing in the early stages by increasing type-I and type-III collagen production. Notably, reduced collagen expression and reduced scar formation was also identified in the late stage (Hu et al., 2016) (Figure 1F). Besides protein and mRNA cargo, noncoding RNAs, particularly microRNAs, have been implicated in the therapeutic effects of EVs in skin wound healing. In one murine incision model, injections of UC-MSC-EVs were found to carry microRNA-27b, which enhanced wound healing *via* the ITCH/JUNB/IRE1α axis (Cheng et al., 2020).

As of now, there are no published results on the use of MSC-EVs in EB patients, and only one clinical trial involving MSC-EVs in EB is currently underway. AGLE-102, an allogenic MSC-EV, is being evaluated in Phase I/IIa for RDEB (NCT04173650).

## 3 Strategies to improve the therapeutic effect of mesenchymal stromal cells in epidermolysis bullosa: author perspective

### 3.1 Delivery approaches of mesenchymal stromal cells

MSCs can be delivered through various routes of administration, including topical application, local injection, and systemic intravenous infusion (Caplan et al., 2019). The choice of administration method is critical to optimizing the therapeutic benefits of MSCs while minimizing potential risks to the patient. Factors such as the pharmacokinetics and pharmacodynamics of the therapy must be considered to determine the most effective approach. Given that certain EB subtypes may have multi-organ manifestations, systemic administration might be advantageous as it allows for broader therapeutic coverage. However, this approach carries additional risks, including infection and bleeding at the injection site. Another strategy is to selectively target affected areas only with regional administration.

#### 3.1.1 Intravenous infusion

Intravenous infusion is the most common delivery method for MSCs (Caplan et al., 2019), however, a large proportion of MSCs that are intravenously delivered accumulate in the lungs - a phenomenon known as the “pulmonary first-pass effect”. This effect is particularly beneficial in treating certain pulmonary conditions, such as acute respiratory distress syndrome (Fischer et al., 2009). Given that certain subtypes of EB, including JEB caused by ITGA3 mutations and severe generalized forms of EB simplex (EBS), involve respiratory complications in addition to mucocutaneous symptoms (Fine et al., 2014), intravenous administration of MSCs may be a promising approach for EB treatment as it has shown early success for other pulmonary conditions, such as acute respiratory distress syndrome (Xiao et al., 2020). Additionally, intravenous infusion of MSCs has shown potential in targeting cutaneous injuries, as demonstrated in studies where BM-MSCs promoted skin vascularization and regeneration in mouse model of mechanically stretched skin (Zhou et al., 2015), and in clinical trials of BM-MSCs in patients with steroid-refractory chronic graft-versus-host disease showing improved skin histopathology (Boberg et al., 2020).

#### 3.1.2 Topical application and local injection

Local injection of MSCs into the skin has been explored as a minimally invasive method of administration in various clinical settings, including burns (Magne et al., 2018) and chronic wounds (Hanson et al., 2010). For example, intradermal injection of Wharton’s jelly-derived MSCs was investigated in patients with alopecia areata under local anesthesia with favorable efficacy and safety outcomes (Czarnecka et al., 2021). While local MSC injections generally result in minimal systemic side effects, direct injection into burns or chronic wounds can be poorly tolerated by patients, sometimes necessitating local anesthesia; this can also be seen in conditions like alopecia areata, which does not involve open wounds (Czarnecka et al., 2021). Likewise, in the context of EB, current treatments involving intradermal injections, such as fibroblast or gene replacement therapy (dabocemagene autoficel or lenticol-F), can cause significant pain due to direct injection into the affected skin. Less invasive topical injection methods, such as intrablister administration, has been shown to be more efficacious than intradermal injections in preclinical investigations of Bf-MSCs (Kikuchi et al., 2023).

Topical preparations of MSCs have been investigated as a less painful alternative for wound treatment. Falanga et al. developed a fibrin polymer-based sprayable formulation of BM-MSCs, which demonstrated significantly accelerated wound healing in preclinical models of acute and diabetic wounds (Falanga et al., 2007). When tested in patients with acute wounds following Mohs micrographic surgery, the MSC spray accelerated wound closure compared to untreated control wounds. The spray also showed remarkable efficacy in treating chronic wounds secondary to diabetes mellitus, including the complete healing of a recalcitrant venous ulcer that had persisted for over a decade. Other non-injectable local delivery systems, such as fibrin matrices (Clover et al., 2015), collagen-fibrin scaffolds (Mirshekar et al., 2023), and fibrin hydrogels (Banerjee et al., 2019), have shown promise in animal models of burns.

Given the allodynia experienced by EB patients (von Bischhoffshausen et al., 2017), topical application of MSCs without injections may be important in the context of EB. Future research could focus on leveraging advances in topical delivery systems in conjunction with MSCs to treat EB. For instance, lattice microarray/microneedle patches (L-MAPs), developed through 3D-printing technology, offer a scalable and precise transdermal delivery method that can painlessly puncture the epidermal or dermal layer (Rajesh et al., 2022). L-MAPs could enable the direct transplantation MSCs into the specific layer of the skin involved in EB while minimizing the intolerable pain associated with local injections.

#### 3.1.3 Other delivery systems

Intraarterial administration of MSCs offers the advantage of reaching deep targets such as the brain and gastrointestinal tract by bypassing the pulmonary first-pass effect (Caplan et al., 2019). This method could be particularly intriguing for EB subtypes with gastrointestinal involvement (Fine et al., 2014). However, the impact on the skin remains unclear. In one case report, intraarterial administration of BM-MSCs for graft-versus-host disease led to partial improvement in the gastrointestinal system, but no data on the skin’s response was provided (Arima et al., 2010). In addition, intravitreal administration of MSCs has been explored in animal studies for conditions affecting the globe and orbit, such as retinitis pigmentosa (Ezquer et al., 2016; Wang et al., 2017), and hence this route might be an interesting option for EB subtypes with ocular involvement (Satarian et al., 2017).

### 3.2 Priming mesenchymal stromal cells

To enhance the therapeutic efficacy of MSCs, various priming strategies have been investigated. These include culturing MSCs under hypoxic conditions (Theus et al., 2008; Tsai et al., 2012), exposing them to ultrasound (Razavi et al., 2020; Liu et al., 2020), culturing them in serum-free conditions (Li et al., 2015; Pachler et al., 2017), and adding specific supplements to the culture medium (Alagesan et al., 2022). Exploring these priming techniques in the context of EB may represent a promising future direction for improving MSC-based therapies (Ezquer et al., 2025).

Culturing cells with pro-inflammatory cytokines is among the most promising priming strategies explored to date, as it has been shown to enhance the efficacy of MSC-based therapies through various mechanisms (Noronha et al., 2019). For instance, research has demonstrated that culturing MSCs with interferon-gamma (IFN-γ) can improve the anti-inflammatory effects of MSCs (Noronha et al., 2019; Alagesan et al., 2022). Duijvestein et al. showed that MSCs primed with INF-γ significantly attenuated dextran sodium sulfate (DSS)-induced colitis in mice, while MSCs cultured in standard conditions did not. Their findings suggest that IFN-γ priming significantly enhances cellular trafficking by upregulating key lectins involved in cell adhesion to epithelial surfaces (Duijvestein et al., 2011). Additionally, Meisel et al. showed that IFN-γ priming increased the production of indoleamine 2,3-dioxygenase (IDO), a key modulator of T-cells, B-cells, and natural killer cells. The induction of IDO also inhibited the growth of *Staphylococcus aureus, Staphylococcus epidermidis, and Toxoplasma gondii* when co-cultured with the primed MSCs (Meisel et al., 2011). The antibacterial properties if IFN-γ primed MSCs could be critical for severe phenotypes, such as RDEB, where the compromised skin barrier predisposes patients to infection, particularly with skin flora (*i.e., Staphylococcus aureus, S. epidermidis*). In pre-clinical studies, priming gingival MSCs with IL-1β modified the MSC secretome, resulting in superior wound healing effects through matrix metalloproteinase and transforming growth factor-β1 (TGF-β1) signaling pathways (Magne et al., 2020).

In addition, the efficacy of MSC priming can be further improved by using a combination of pro-inflammatory cytokines rather than a single cytokine (Noronha et al., 2019; Alagesan et al., 2022). Horie et al. found that MSCs primed with a cytokine cocktail including TNF-a, interleukin-1 beta (IL-1β), and IFN-γ significantly improved lung compliance and oxygenation in a murine model of ventilator-induced lung injury compared to non-primed MSCs (Horie et al., 2020). Similarly, Byrnes et al. showed that the same cytokine cocktail enhanced oxygenation and lung compliance in an antimicrobial-resistant model of *Klebsiella pneumoniae*-induced pneumonia in rats more effectively than naïve MSCs (Byrnes et al., 2023).

### 3.3 Induced pluripotent stem cell-derived mesenchymal stromal cells

Another promising approach in MSC-based therapies is the use of induced pluripotent stem cell (iPSC)-derived MSCs (i-MSC) (Hynes et al., 2013). A significant challenge in scaling up MSC-based therapies is the finite proliferative capacity of MSCs and their tendency to undergo spontaneous mutations after repeated passaging, which can impede their therapeutic efficacy. Furthermore, MSCs display widely variable properties depending on the donor and the tissue source from which they are harvested (Zhao and Ikeya, 2018). These challenges can be largely addressed by using MSCs derived from iPSCs. Somatic cells can be isolated from any tissue source within the body and reverted into a pluripotent state through culture with particular transcription factors, thereby providing a readily available source of iPSCs with minimal ethical concerns (Hynes et al., 2013). Several protocols have been developed to differentiate iPSCs into MSC-like cells, which retain the therapeutic potential of conventional MSC while mitigating issues related to donor variability and limited proliferative capacity (Fukuta et al., 2014; Hynes et al., 2014; Lian et al., 2016).

Soontararak et al. conducted a comparative study between AD-MSCs and i-MSCs for treating DSS-induced colitis model in mice. Their results indicate no significant difference in gross lesion scores or histological inflammation scores between the two treatment groups. This suggests that i-MSCs are as effective as AD-MSCs in this colitis model (Soontararak et al., 2018). In a separate study, Lian et al. evaluated the effectiveness of i-MSCs for the treatment of limb ischemia in mice. The study demonstrated that i-MSCs were more effective in attenuating hind limb ischemia than BM-MSCs. Notably, the incidence of limb loss in the i-MSC group was less than half of those who suffered limb loss in the BM-MSC group (Lian et al., 2010). Similarly, Sun et al. investigated the effects of i-MSCs in a mouse model of ovalbumin-induced allergic airway inflammation. The study found that i-MSCs led to reductions in peribronchial inflammatory cell infiltration, goblet cell hyperplasia, and nasal submucosal eosinophilia, comparable to those achieved with BM-MSCs (Sun et al., 2012). Taken together, these studies highlight that the promise of i-MSCs in overcoming the variability and limitations associated with traditional MSCs. However, non-inferiority trials specifically for EB will be necessary before these findings can be translated into clinical practice.

### 3.4 Genetic engineering of mesenchymal stromal cells

Genetic modification of MSCs has gained attention as significant area of interest due to its potential to precisely tune MSC properties. For example, AD-MSCs have been engineered to overexpress tumor necrosis factor-related apoptosis-inducing ligand (TRAIL) and TNF-α, resulting in reduced tumor progression in non-small cell lung cancer mouse models by inducing apoptosis in cancer cells (Choi et al., 2021; Snajdauf et al., 2021). In murine models of peritoneal carcinomatosis, BM-MSCs were modified to overexpress IL-12 and IL-21, showing greater therapeutic efficacy when compared to anti-PD1 checkpoint inhibitors (Gonzalez-Junca et al., 2021). In the context of RDEB, Petrova et al. used lentiviral transduction to genetically modify UC-MSCs to express C7. They found that intradermal injection of these engineered cells into EB skin grafts in mice led to C7 deposition at the DEJ, and promoted the formation of *de novo* anchoring fibril, a critical component for skin integrity in RDEB patients (Petrova et al., 2020). These studies underscore the potential of genetically modified MSCs to extend beyond traditional applications, offering novel and customizable therapeutic options for EB.

## 4 Conclusion

EB is a complicated, heterogeneous group of genodermatoses for which current treatments are not curative and face several limitations. Gene-based therapies have been at the forefront of recent research; however, current treatments are limited to small body surface areas. Additionally, gene editing therapies pose challenges due to the potential for off-target effects.

MSCs are being investigated as therapeutic agents across a wide array of pathologies, including preliminarily in RDEB, where they have been shown to be safe and effective. MSCs are a fascinating area of research for EB treatment because of their success in chronic wound healing and various other conditions, suggesting that MSC-based therapy could be effective across multiple EB subtypes, regardless of underlying mutations. This hypothesis is also supported by the evidence of a C7-independent pathway in RDEB studies (Webber et al., 2017).

Recent multiple early phase trials of MSCs for the treatment of EB show an encouraging safety profile. Additionally, MSCs have demonstrated clinical improvement in EB, though these effects are transient. To optimize MSC treatment for EB, including the length of treatment effect, future clinical trials should investigate the various methods of modifying MSC therapy, one of its most appealing properties. As reviewed in our work, MSCs can be primed in various ways, such as culturing with cytokine cocktails, to boost their efficacy. Moreover, the use of MSC-derived EVs, which contain bioactive molecules capable of modulating cellular responses, offers a cell-free alternative that could mitigate some of the risks associated with live cell therapies, such as tumorigenicity or immune rejection. Advances in delivery systems also hold promise for improving the efficacy and accessibility of MSC-based therapies for EB patients. Topical and transdermal applications, for example, could provide direct and localized delivery of MSCs or MSC-derived products to affected skin areas, enhancing treatment efficacy while minimizing systemic side effects and reducing the discomfort associated with injections. Additionally, these delivery innovations could make MSC therapies more accessible, less invasive and patient-friendly, ultimately improving the quality of life for individuals living with EB.

Critically, there is a need for harmonized trial design and standardized endpoints to allow for cross-study comparison for these different approaches to MSC therapy, as current trial designs use heterogenous endpoints that make comparisons difficult. Furthermore, MSCs could serve to be powerful adjunctive therapies to the changing landscape of EB therapy, such as in combination with beremagene gerperpavec (Vyjuvek) by decreasing inflammation and enhancing wound healing.

However, several questions remain regarding the use of MSCs as a therapy for treating EB. The precise mechanism by which MSCs exert their therapeutic effects are not fully understood, and it is unclear whether the C7-independent benefits are solely due to anti-inflammatory actions or involve other factors. Additionally, the efficacy of MSCs derived from different tissue sources needs further exploration.

In conclusion, while MSC-based therapies represent a promising avenue for treating EB, further research is needed to fully understand their mechanisms, optimize their efficacy, and develop robust delivery systems. Addressing these challenges will be crucial for advancing MSCs from experimental therapies to clinically viable treatments that offer hope for patients with EB.
